# Research Progress on the Oxidation Behavior of Ignition-Proof Magnesium Alloy and Its Effect on Flame Retardancy with Multi-Element Rare Earth Additions: A Review

**DOI:** 10.3390/ma17133183

**Published:** 2024-06-28

**Authors:** Duquan Zuo, Haolin Ding, Maoyong Zhi, Yi Xu, Zhongbo Zhang, Minghao Zhang

**Affiliations:** 1 Civil Aircraft Fire Science and Safety Engineering Key Laboratory of Sichuan Province, Civil Aviation Flight University of China, Guanghan 618307, China; 2 College of Aviation Engineering, Civil Aviation Flight University of China, Guanghan 618307, Chinazbzhang2001@163.com (Z.Z.); 3 School of Mechanical Engineering, Northwestern Polytechnical University, Xi’an 710072, China

**Keywords:** ignition-proof magnesium alloy, rare earth, flame retardancy, oxidant behavior

## Abstract

The phenomenon of high-temperature oxidation in magnesium alloys constitutes a significant obstacle to their application in the aerospace field. However, the incorporation of active elements such as alloys and rare earth elements into magnesium alloys alters the organization and properties of the oxide film, resulting in an enhancement of their antioxidation capabilities. This paper comprehensively reviews the impact of alloying elements, solubility, intermetallic compounds (second phase), and multiple rare earth elements on the antioxidation and flame-retardant effects of magnesium alloys. The research progress of flame-retardant magnesium alloys containing multiple rare earth elements is summarized from two aspects: the oxide film and the matrix structure. Additionally, the existing flame-retardancy models for magnesium alloys and the flame-retardant mechanisms of various flame-retardant elements are discussed. The results indicate that the oxidation of rare earth magnesium alloys is a complex process determined by internal properties such as the structure and properties of the oxide film, the type and amount of rare earth elements added, the proportion of multiple rare earth elements, synergistic element effects, as well as external properties like heat treatment, oxygen concentration, and partial pressure. Finally, some issues in the development of multi-rare earth magnesium alloys are raised and the potential directions for the future development of rare earth flame-retardant magnesium alloys are discussed. This paper aims to promote an understanding of the oxidation behavior of flame-retardant magnesium alloys and provide references for the development of rare earth flame-retardant magnesium alloys with excellent comprehensive performance.

## 1. Introduction

Magnesium alloys, recognized for their low density, high specific strength, and excellent impact resistance, have garnered significant favor within the aviation manufacturing sector [1,2,3]. However, magnesium alloys exhibit poor deformability and ductility and are prone to oxidation at high temperatures [4]. The oxidation of magnesium alloys leads to a heightened risk of ignition and subsequent combustion, posing significant safety concerns. Their high affinity for oxygen renders them incapable of self-extinguishing, resulting in the prohibition of magnesium alloys in aircraft applications [5]. It was not until the Federal Aviation Administration (FAA) conducted an eight-year safety assessment of magnesium alloys that the ban on their use within aircraft cabins was lifted. This indicates the potential feasibility of employing magnesium alloys in civil aviation, especially within cabin interiors, provided advancements in ignition resistance and oxidation mitigation are achieved [6,7]. Hence, comprehensively understanding the oxidation and ignition behaviors of magnesium alloys at high temperatures and developing alloys resistant to high-temperature oxidation and combustion are crucial steps in broadening the application scope of magnesium alloys within the civil aviation domain.

Combustion is a specific process of high-temperature oxidation, representing an intense form of oxidative behavior [8]. Researchers have discovered that rare earth (RE) elements, when used as alloy constituents, exhibit favorable solubility within the α-Mg matrix, offering an effective flame-retardant method to enhance the ignition point and resistance to high-temperature oxidation of magnesium alloys [9,10]. RE elements mainly comprise the Ce group and the Y group, with the former being light rare earths (LREs): La to Eu, and the latter being heavy rare earths (HREs): Gd to Y [11,12,13]. In the α-Mg matrix, the solubility of the LREs is lower than that of the HREs. This paper provides a comprehensive review of the influencing factors on flame-retardant magnesium alloy high-temperature oxidation processes, with a specific focus on summarizing the research progress on the surface oxidation behavior of multi-element rare earth flame-retardant magnesium alloys, encompassing existing flame-retardancy models and the flame-retardant mechanisms of various alloying elements.

### 1.1. Investigation of Low- and High-Temperature Oxidation Behavior of Magnesium Alloys

**Figure 1:**
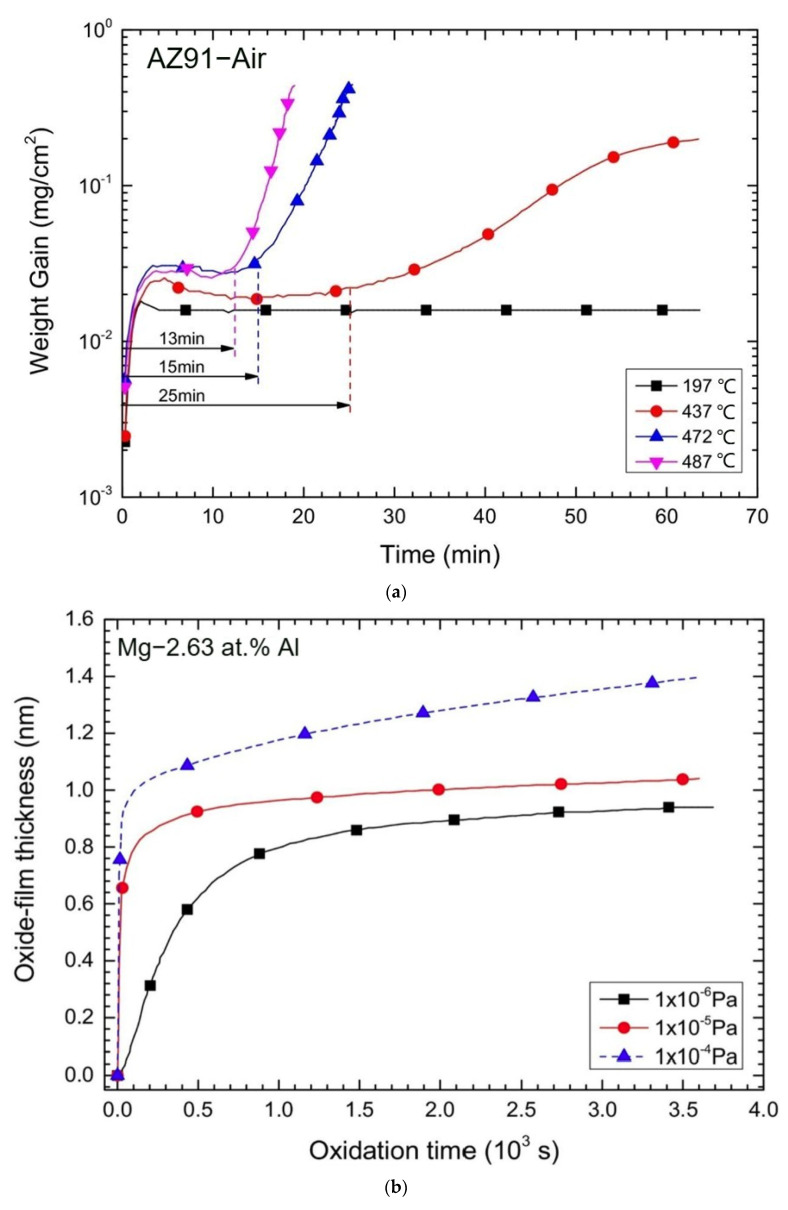
The thermogravimetric measurements of weight gain versus time for as-cast magnesium alloy in air at different temperatures [14]. (**a**) Thermogravimetric curve of AZ91 in air over time, and (**b**) relationship between oxide film thickness and oxidation time of Mg-2.63 at.% Al at 31 °C and indicated oxygen pressure.

Magnesium alloys possess high chemical reactivity and are highly prone to oxidation and subsequent combustion at high temperatures [4,8]. High-temperature oxidation of magnesium alloys follows a parabolic characteristic [14], as depicted in Figure 1a, comprising incubation and accelerated oxidation stages. Within a certain temperature range, magnesium alloys exhibit a certain resistance to oxidation, with an accelerated oxidation stage occurring only beyond a critical temperature, typically ranging between 400 °C and 450 °C [15]. Thermal analysis reveals that below 400 °C (corresponding to curve 197 °C), only an incubation period is observed, with minimal oxidation enhancement even after 13 min, indicating the formation of a highly dense MgO layer on the magnesium alloy surface capable of shielding the substrate from further oxidation. Beyond 400 °C, there is a significant increase in the oxidation rate, leading to the loss of the protective capabilities of the MgO layer. As the temperature escalates from 437 °C to 487 °C, the incubation period diminishes from 25 min to 13 min, signifying a notable decrease in oxidation resistance. Studies also demonstrate [16,17,18] that with rising temperatures, the high-temperature oxidation incubation period of magnesium alloys drastically shortens, prompting an early transition into the accelerated oxidation stage. Hence, it can be concluded that enhancing the resistance of magnesium alloys to high-temperature oxidation or flame retardance essentially involves prolonging the high-temperature oxidation incubation period of magnesium alloys.

### 1.2. Low-Temperature Oxidation below 400 Degrees

Figure 1b depicts the kinetic oxidation curve of magnesium alloy at 31 °C under low oxygen pressure. Initially, the growth of the oxide film is rapid, with higher oxygen pressures resulting in elevated growth rates and shorter durations [19]. Subsequently, the growth rate stabilizes independent of oxygen pressure, indicating that the variation in oxide film thickness follows the parabolic law, where the formed MgO oxide layer segregates the reactants and governs the oxidation rate through material transport within the MgO oxide layer [20]. Hence, it is inferred that at low temperatures, the MgO layer acts as a compact thin film capable of shielding the substrate from oxidation.

### 1.3. High-Temperature Oxidation above 400 Degrees

**Figure 2:**
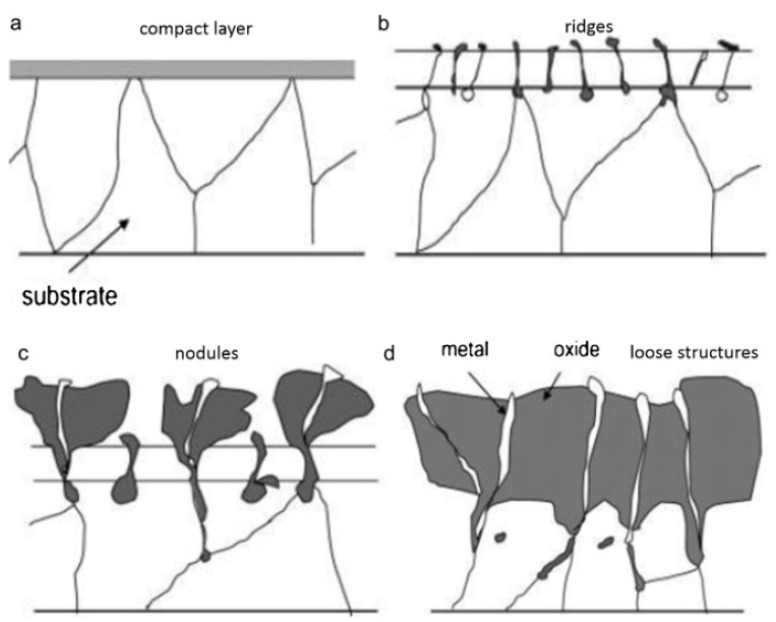
Schematic of high-temperature oxidation and burning of magnesium alloy [14]. (**a**) a compact layer showing protective properties; (**b**) cracking of the layer and growth of oxide ridges; (**c**) nodular growth accompanied with linear kinetics; (**d**) coalescence of nodules and the formation of loose layer with pores.

The high-temperature oxidation of magnesium alloys involves multiple processes such as Mg vapor and Mg^2+^ ion lattice diffusion, culminating in the accumulation of stress within the oxide layer, leading to its rupture and subsequently accelerating Mg oxidation [14]. Figure 2 illustrates the high-temperature oxidation process and combustion mechanism of magnesium alloys [19,20,21]. In the incubation stage of high-temperature oxidation (see Figure 2a), a dense and continuous oxide layer forms on the surface of magnesium alloys. This dense layer isolates the substrate from oxygen, providing a certain period of protection for the magnesium alloy [13,15,22]. As the oxidation reaction progresses, Mg^2+^ diffuses outward through the oxide layer, creating voids at the metal/oxide interface and generating localized stress. Simultaneously, the high-temperature vapor of magnesium gathering beneath the oxide layer increases localized pressure. The combined action of these two sources of localized stress leads to the rupture of the oxide layer [22,23]. The appearance of cracks in the oxide layer also signifies the end of the incubation stage of magnesium alloy oxidation and the onset of accelerated oxidation. Figure 2b depicts the cracking and spine formation of MgO layers. During growth, magnesium vapor, using the pathways formed by these cracks, reacts with O_2_ at the alloy/oxide interface, promoting spine outward growth. MgO deposits and nucleates accordingly [24,25]. As shown in Figure 2c, with the increasing thickness of the oxide layer, the initially formed MgO layer transforms from a smooth, continuous form to a protruding nodular oxide. Furthermore, with an increase in the number of cracks in the oxide layer, the oxidation rate significantly accelerates linearly, as shown by the blue curve (corresponding to 472 °C) and the pink curve (corresponding to 487 °C) in Figure 1a.

In summary, MgO films, below the critical temperature, serve as a protective oxide to prevent the substrate from oxidation, resulting in a slow growth of the oxide film. However, beyond the critical temperature, the oxidation process involves two stages: the incubation stage and the accelerated oxidation stage. The former follows a parabolic kinetic oxidation, while the latter adheres to linear growth kinetics. Furthermore, at higher temperatures, magnesium alloys transition earlier into the accelerated oxidation stage, leading to a shorter incubation period.

## 2. The Fire Combustion Process

One characteristic of Mg combustion is the substantial release of heat and the generation of bright visible light [26]. Under high-temperature conditions, oxidative reactions easily lead to the ignition and combustion of magnesium alloys [27,28]. As depicted in Figure 2d, when magnesium and oxygen diffuse without restraint, a rapid reaction occurs between magnesium vapor and O_2_, forming oxide islands that continuously accumulate, resulting in a porous and loose oxide layer. At elevated temperatures, when the input heat surpasses the heat consumed by radiation and thermal conduction of the magnesium alloy, oxidative reactions intensify, eventually creating conditions conducive to ignition and combustion [26]. Czerwinski et al. [29,30] investigated a series of magnesium alloy combustion issues and found that in all experiments, with increasing temperatures, significantly noticeable Mg vapor exists in the semi-solid or molten state of magnesium alloys. Liquid or gaseous magnesium vapor continuously diffuses outward through cracks in the oxide layer, saturating the porous oxide and leading to ignition and combustion.

The combustion of magnesium is accompanied by the evaporation of Mg and the generation of a substantial amount of heat; hence, magnesium alloys are considered a type of metal material that sustains combustion and is not easily extinguished [5,14,29]. Wright et al. [31] characterized magnesium alloys by their combustibility, indicating a continued tendency for combustion even after the removal of the flame.

**Figure 3:**
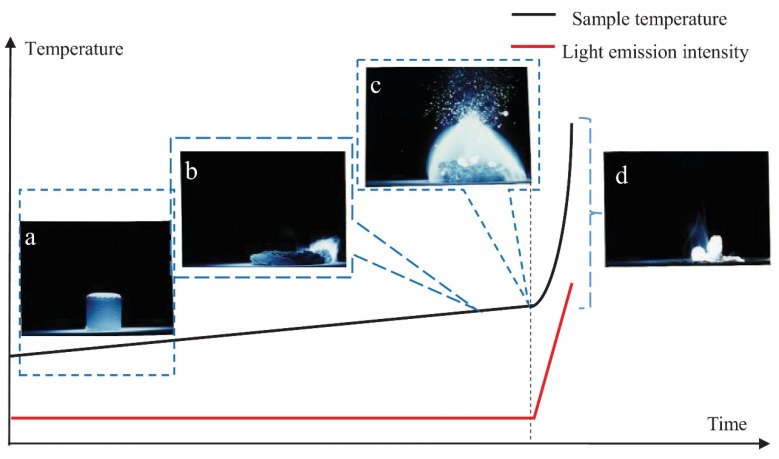
Schematic of temperature and luminescence intensity of magnesium alloy with time [32]. (**a**) Formation of MgO Outer Layer; (**b**) Melting and Expansion of Mg Sample; (**c**) Increase in Vapor Pressure and Rupture of MgO Layer; (**d**) Intense Oxidation and Visible Flame.

Figure 3 illustrates the entire process of Mg sample combustion from the solid phase to complete combustion, depicting the variation in luminosity over time [32]. Before reaching a temperature below the melting point of 650 °C, a white substance, MgO, is present on the outer layer of the Mg sample (refer to Figure 3a). As the temperature raises towards the melting point, the interior of the solid Mg sample gradually starts to melt and expand (as depicted in Figure 3b). Subsequently, magnesium vapor accumulates within the Mg sample, causing an increase in vapor pressure until the outer MgO layer ruptures (see Figure 3c). At this juncture, a substantial amount of Mg and Mg vapor undergoes oxidation, generating a visible, intense flame (see Figure 3d). Studies indicate that during the combustion process of magnesium alloys, if the generated heat is insufficient and the oxide film remains intact and dense, the mass and oxygen transport capability decline, potentially leading to non-ignition and combustion failure [32,33,34].

## 3. Analysis of Ignition- and Flame Retardancy-Influencing Factors

The primary factors influencing the ignition characteristics of magnesium alloys encompass not only the intrinsic attributes of the samples but also experimental conditions such as oxygen concentration, partial pressure, temperature variation rates, and test methodologies [35,36]. Current research endeavors assessing the antioxidation and combustion resistance of flame-retardant magnesium alloys invariably involve ignition point evaluations. Despite variations in the geometrical shapes of magnesium alloys utilized across different test methodologies [35,37] and disparate testing conditions [32,36,38,39], the resulting data often exhibit significant disparities. Nonetheless, discussions concerning antioxidation and combustion mechanisms tend to converge on certain common conclusions. Accordingly, this section focuses on delineating and analyzing the impact of alloying elements, solid solubility, and intermetallic compounds (second phases) on the antioxidative properties and flame-retardant efficacy of magnesium alloys.

### 3.1. Alloying Elements

Alloying has garnered significant attention as a crucial method for enhancing various properties of alloys. In the case of magnesium alloys, the addition of suitable surface-active elements, combined with oxygen, leads to the formation of a dense magnesium oxide film on the alloy’s surface, significantly improving the high-temperature resistance of magnesium alloys. LEE et al. [40] discovered that at elevated temperatures, Ca-containing flame-retardant magnesium alloys can enhance flame resistance by forming a dense CaO-rich outer layer and a MgO/CaO inner oxide film, preventing contact with O_2_. In addition to generating a protective oxide film, Ca improves the oxidation resistance of magnesium alloys by forming a secondary phase with other alloying elements [21,40]. However, the incorporation of alloying elements in the form of oxides rather than elemental forms—a method known as indirect alloying—also significantly impacts the flame resistance of magnesium alloys. Using stable oxide forms of alloying elements, such as CaO, the ECO-Mg technology has been developed by LEE et al. [41]. This technology involves the addition of CaO, resulting in a magnesium alloy called “Environment-Conscious Magnesium (ECO-Mg)”, which favors the formation of a protective mixed oxide film composed of MgO and CaO at high temperatures. As the CaO content increases, the ignition temperature of the alloy rises. Additionally, including CaO reduces the need for greenhouse gases during processing [42]. The protective capability of oxides formed on metals can be represented by the Pilling–Bedworth ratio (PBR), which denotes the ratio between the volume of formed magnesium oxide molecules and the consumed volume of metal atoms, reflecting the stress state within the magnesium oxide film [43,44]. When the PBR is less than 1, the oxide layer formed on the metal surface fails to provide sufficient protection to the substrate. PBR values ranging from 1 to 2 indicate moderate compressive stress in the surface oxide layer of magnesium alloys, characterized by a continuous, dense structure and a high ignition point., signifying its protective role. However, when the PBR exceeds 2, growth stress leads to cracking and delamination of the oxide layer, rendering it incapable of shielding molten magnesium alloys from further oxidation [45,46]. Research indicates that a PBR of 0.73 (<1) in a magnesium oxide film [45,47] signifies substantial internal tensile stress within the oxide film, resulting in a loose structure. Additionally, at a PBR of 1.4 [48], magnesium ions can react with surface oxygen through a porous oxide film, compacting the layer and effectively protecting the magnesium alloy. Thus, the flame-retardant properties of magnesium alloys are closely linked to the nature of the oxide film during the oxidation process and subsequent combustion behavior [49].

**Figure 4:**
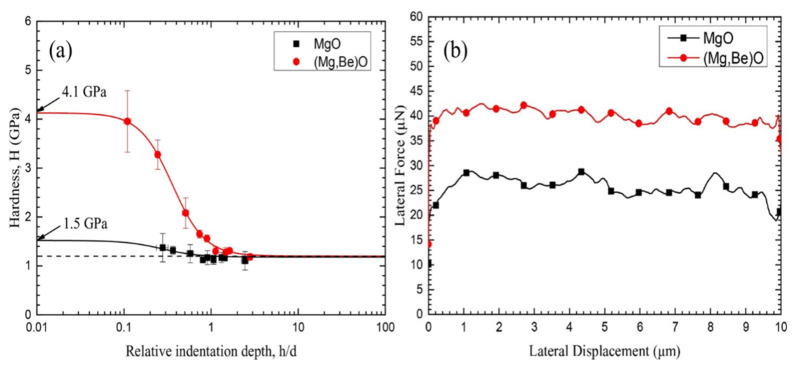
Mechanical properties of the oxide film for AZ91D magnesium alloy with Be element added [53]. (**a**) The relationship between hardness H of each oxide film and relative indentation depth h/d, and (**b**) lateral force of each oxide film as a function of the displacement in nanoindentation test.

At present, the Pilling–Bedworth Ratio (PBR) model [50,51] is commonly employed to characterize the high-temperature oxidation resistance and flame-retardant mechanisms of most magnesium alloys, except Mg-Sr and Mg-Be alloy systems. For instance, in the case of Mg-Sr alloys, based on the aforementioned theory, when the PBR of SrO is 0.66 < 1, a dense oxide film cannot form to protect the magnesium alloy from oxidation [52]. However, due to the presence of the Sr element, the ignition point of the magnesium alloy is elevated, significantly enhancing its oxidation resistance and flame-retardant effect. Concerning Mg-Be alloys, as depicted in Figure 4 [53], the hardness and shear force of the (Mg, Be)O composite oxide film are notably higher than MgO, enabling Be to act as a solid solution strengthening agent and grain refiner within the composite oxide film. Consequently, the (Mg, Be)O composite oxide film exhibits higher resistance to cracking, elevating the ignition temperature of the AZ91D alloy with the added Be element. According to Ouyang et al. [54], apart from considering the impact of oxide film density, flame-retardant effects should also account for the influence of the oxide film on O_2_ permeability. Aydin et al. [55] similarly concluded in their study that the oxide film’s poor O_2_ permeability delays the oxidation of the alloy matrix.

### 3.2. Solid Solubility

**Figure 5:**
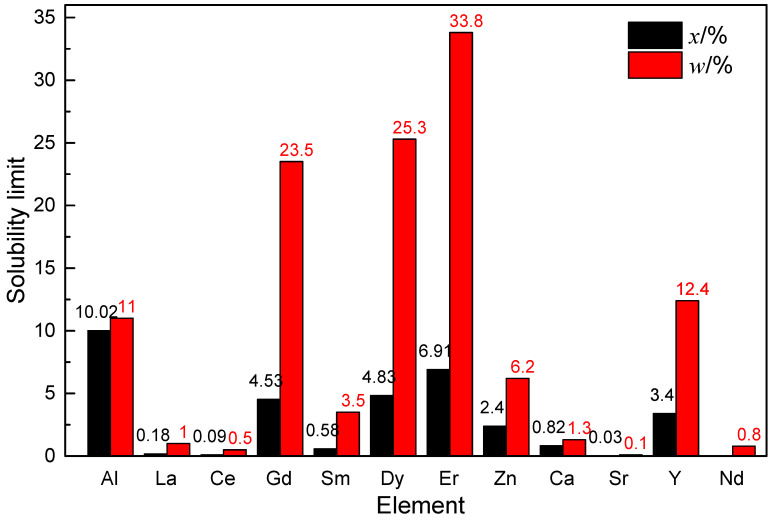
Histogram of solid solubility limits for Mg with the different alloying elements added. Reproduced from [35] with permission from Elsevier.

The flame-retardant properties of alloys are directly proportional to the variation in solute solid solubility within their matrices [35,56]. Figure 5 illustrates the solubility limits of different elements in Mg [35,57,58,59]. Owing to the relatively low solute solubility elements present in the Mg matrix, its composition closely resembles that of pure Mg. At elevated temperatures, highly reactive alloying elements form a dense protective oxide layer primarily composed of MgO. As depicted in Equation (1), this represents the typical reaction between Mg and the active alloying element *X* upon contact at room temperature [9,14]:

$$aMgO+bX\to X_{b}O_{a}+aMg$$

**Figure 6:**
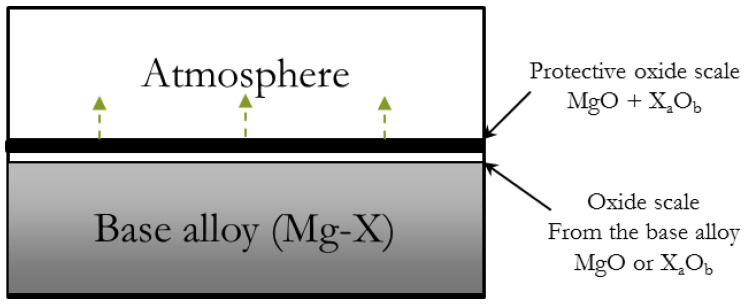
Mechanisms of oxidation at the surface of the base alloy and effect of eutectic phase on oxidation behaviors that suppress ignition of Mg [36].

In the case of stable eutectic-phase alloys, ignition primarily initiates from the matrix exposed to the atmosphere. The formed eutectic phase (Mg + solute atoms) facilitates the diffusion of solute atoms necessary for the formation of a dense MgO/*X*_a_O_b_ oxide layer, aiding in retarding the reaction depicted in Figure 6 [14,36,60,61]. This enhancement contributes to the oxidation resistance of the matrix and prevents ignition of the exposed matrix. Consequently, it can be concluded that solute atoms with notably high solubility limits at elevated temperatures, such as Er, Y, and Gd [36,62], result in a fully solute-saturated matrix exhibiting excellent fire resistance. The rationale behind this lies in the easier formation of a uniform and compact oxide layer at high temperatures.

### 3.3. Intermetallic Compounds (Second Phase)

In addition to the solute concentration of alloying elements in the Mg matrix and the oxide’s PBR (Pilling–Bedworth Ratio) effect, intermetallic compounds play a significant role in the ignition combustion of magnesium alloys. Typically, high-temperature oxidation of alloys leads to microstructural alterations and the melting of secondary phases, thereby lowering the alloy’s ignition point [63,64]. Simultaneously, the formation of porous oxide layers and cracks is influenced by the higher vapor pressure of low-melting point phases, accelerating the rate of protective oxide layer rupture [65]. The combined action of these factors expedites the alloy’s oxidation process. According to Tan et al. [14,21], alloying elements such as Al and Zn correspond to PBR values between 1 and 2 for oxides. Upon alloying with Mg, the formation of the β-Mg_17_Al_12_ phase with a low melting point of 437 °C occurs. With increasing Mg_17_Al_12_ content, the alloy’s melting temperature decreases, facilitating the transition from solid-state combustion to liquid-state combustion, making magnesium alloys more prone to vaporization and ignition. Similarly, the metallic compounds of alloying elements such as Zn, Ca, Ce, Gd, and Sr, with respective melting points of Mg_2_Zn_3_ (614 K), Mg_2_Ca (987 K), Mg_12_Ce_2_ (889 K), Mg_5_Gd (931 K), and Mg_2_Sr (879 K), are formed as a byproduct of the eutectic characteristics of magnesium alloys. This formation can lead to severe evaporation and selective oxidation of the alloy at high temperatures [66].

**Figure 7:**
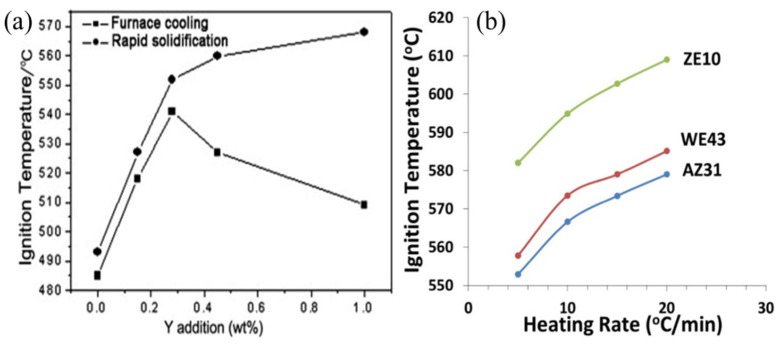
Effect of rapid solidification with Y element added and heating rate on ignition temperature for the different magnesium alloys. (**a**) Ignition temperature of AM50-Y addition after rapid solidification treatment [13,70]. (**b**) Variation of ignition temperature with the heating rate for ZE10, WE43, and AZ31 magnesium alloys [36].

Furthermore, concerning the high melting point second phase, Huang and Kazenas [67,68] discovered that alloying elements, during the melting process of the second phase, sublimate at high temperatures, thereby accelerating the formation of cracks and voids on the oxide layer, leading to the deterioration of the oxide layer. Li [38] and Yu et al. [69] separately substantiated the adverse effects of the high-temperature stable phases Al_11_Ce_3_ and A_l2_Y. Their formation and growth consume substantial amounts of Ce and Y within the matrix, resulting in regions impoverished of alloying elements. Consequently, insufficient alloying elements are available to form Ce_2_O_3_/CeO_2_ (PBR = 1.14/1.15) oxide layers and Y_2_O_3_ (PBR = 1.13) oxide layers, thereby reducing the alloy’s resistance to high-temperature oxidation. Simultaneously, Ni et al. [13] and Lin et al. [70] investigated the influence of cooling rate on the ignition combustion and flame-retardant properties of the AM50-Y alloy. It is observed that rapid solidification treatment can enhance the alloy’s flame retardancy by altering the distribution and composition of the second phase, as depicted in Figure 7. This is due to the excessively high cooling rate in rapid solidification, which prevents the formation of high-melting point phases, thereby increasing the solubility of Y in the Mg matrix, nullifying the influence of the second phase and enabling sufficient Y atoms to form a protective film, thus enhancing flame retardancy. Consequently, it can be concluded that rapid solidification effectively inhibits the formation of high-melting point phases, extends solubility, and forms a uniform microstructure to enhance oxidation resistance and flame retardancy.

Subsequently, Tekumalla et al. [36] conducted similar work on heating rates (see Figure 7b), investigating the relationship between heating rates and ignition temperatures for different test materials. Their findings revealed that slower heating rates yielded more conservative results compared to faster heating rates, with the ZE10 alloy demonstrating superior flame-retardant properties. Additionally, the oxidation of certain alloys predominantly occurred in the second phase and increased [61,71], leading to a decline in the alloy’s resistance to high-temperature oxidation and flame retardancy. This conclusion suggests that the oxidation sequence of the alloy is another factor influencing the formation of the second phase.

In summary, the ignition and combustion process of magnesium alloys is intricately complex, with the relationship between high-temperature oxidation resistance and flame retardancy being influenced by various factors. Understanding these aspects often requires a comprehensive consideration of the interrelatedness among these factors and their resultant impacts. However, current research predominantly focuses on singular factors to explore the influencing elements of high-temperature oxidation and flame retardancy in magnesium alloys, overlooking the holistic perspective. It is evident that to accurately assess the high-temperature oxidation resistance and flame retardancy of magnesium alloys, elucidating their mechanisms and establishing quantitative relationships between these mechanisms and their high-temperature oxidation and flame-retardant properties necessitates deeper investigation. Further refinement in theory is required.

## 4. The Influence of Diverse Rare Earth Elements on the Flame Retardancy of Magnesium Alloys

**Figure 8:**
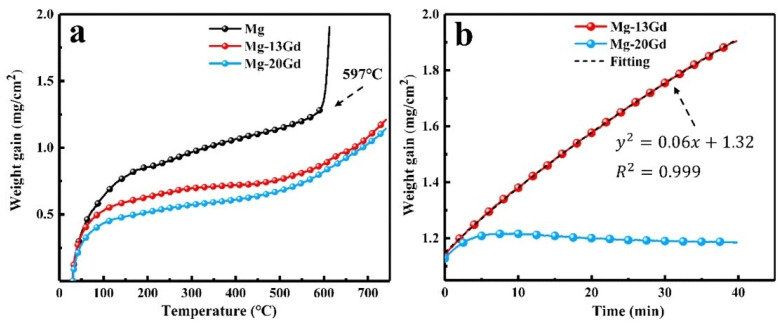
Thermogravimetric analysis curves [77]: (**a**) Oxidation dynamic curves at a rate of 283 K/min, and (**b**) oxidation dynamic curves at 1013 K.

As previously mentioned, alloying with small amounts of elements such as Ca, Be, and Sr is an effective method to enhance the antioxidation capability of magnesium alloys. However, improper addition of Ca and Be can adversely affect the mechanical properties and corrosion resistance of the alloy [72,73,74], and the toxicity of Be restricts the practical application of flame-retardant magnesium alloys [75]. Nevertheless, individual rare earth elements have the capability to refine the grain structure of magnesium alloys, thereby improving their room- and high-temperature formability, strength, heat resistance, and corrosion resistance. This has led to rare earth magnesium alloys becoming a focal point in current research on flame-retardant materials [76]. According to reports by Cai et al. [77], a study was conducted on the high-temperature oxidation kinetics of Mg-xGd alloys with high Gd content. The oxidation dynamic curves at a heating rate of 283 K/min and a temperature of 1013 K are presented in Figure 8. As depicted in Figure 8a, the weight gain curves of Mg-13Gd and Mg-20Gd do not show a rapid increase between 303 K and 1013 K. However, the pure Mg alloy undergoes intense oxidative combustion at 870 K, indicating a vigorous oxidation reaction. Observing Figure 8b, it is evident that the Mg-20Gd alloy exhibits superior oxidation resistance at 1013 K compared to the Mg-13Gd alloy (the weight gain curve of the former is remarkably flat). Furthermore, the fitted results of the weight gain curves show a parabolic shape, indicating that the growth mode of the oxide film is an ideal protective oxidation process. The dense oxide layer isolates the oxidizing medium from the substrate, playing a role in antioxidation and fire resistance with the density of the oxide film approaching 1 [78].

Further investigation revealed that the widely used AZ alloy did not meet the standards in flammability tests, while the WE43 alloy, containing multiple rare earth elements, formed a dense Y_2_O_3_ oxide film during testing, demonstrating excellent flame-retardant properties and successfully passing the tests [30,36]. Additionally, the appropriate addition of various rare earth elements can significantly improve the oxidation layer, allowing rare earth magnesium alloys to exhibit higher ignition points and antioxidative properties [2]. Therefore, this section will focus on the impact of binary and ternary rare earth elements on the flame retardancy of magnesium alloys.

### 4.1. The Impact of Binary Rare Earth Elements on the Flame Retardancy of Magnesium Alloys

#### 4.1.1. Y and Gd

**Figure 9:**
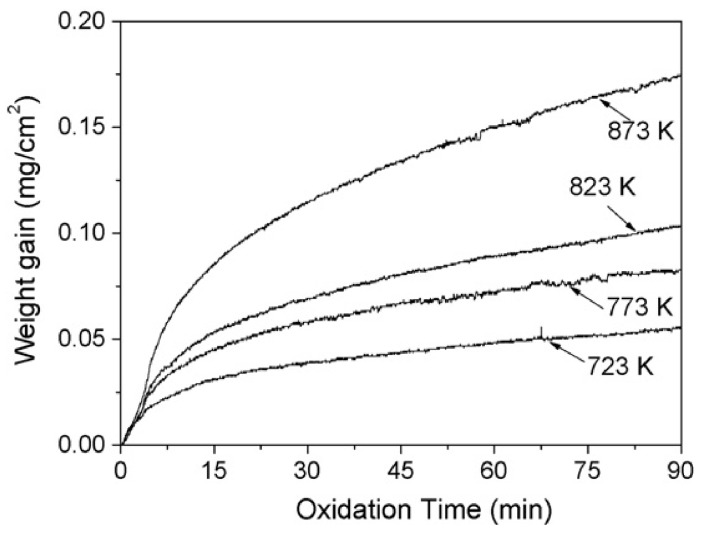
Weight gain curves of the Mg-10Gd-3Y alloys oxidized in pure O_2_ at different temperatures up to 90 min [79].

Yttrium (Y) and Gadolinium (Gd), as rare earth elements, exhibit favorable effects in enhancing the flame retardancy of magnesium alloys. According to reports by Wang et al. [79], under conditions of a heating rate of 50 K/min and an oxygen flow rate of 60 mL/min, an isothermal oxidation experiment lasting 90 min was conducted on the Mg-10Gd-3Y alloy. The oxidation dynamic curve of this alloy is depicted in Figure 9. Upon observing Figure 9, it is evident that the alloy experiences the most significant weight gain at 873 K during the same time interval. The curve assumes a parabolic shape, indicating an increase in oxidation layer thickness with the rise in oxidation temperature, resulting in weight gain. Concurrently, a dense oxide film composed of Gd_2_O_3_, Y_2_O_3_, and minor MgO is formed, which retards the internal diffusion of oxygen, thereby enhancing the oxidation resistance of the substrate.

**Figure 10:**
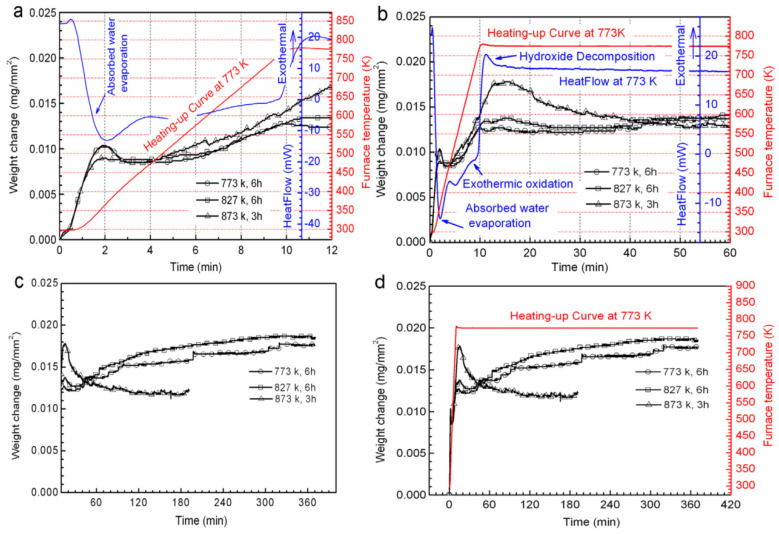
Results of thermogravimetric measurements for the alloy oxidized in pure O_2_ [80]. (**a**) Initial Rapid Weight Gain and Water Evaporation; (**b**) Weight Loss Due to Hydroxide Decomposition; (**c**) Parabolic Kinetics and Continuous Weight Increase; (**d**) Weight Peak and Subsequent Stabilization.

Wu et al. [80] discovered that the oxidation behavior of the Mg-10Gd-3Y alloy bears similarity to that of the Mg-2.1Gd-1.1Y-0.82Zn-0.11Zr alloy, both following a parabolic kinetic law. Figure 10 presents the thermogravimetric analysis results of the Mg-2.1Gd-1.1Y-0.82Zn-0.11Zr alloy at three different temperatures. As shown in Figure 10a, before 2 min, there was a rapid increase in alloy weight due to the oxidation of Y, Gd, Mg, and Zn, forming oxide layers of MgO, Y_2_O_3_, Gd_2_O_3_, and ZnO, among others. Between 2 to 4 min, there was an incremental rise in alloy weight primarily caused by water molecules. Subsequently, during the isothermal linear oxidation stage (4 to 10 min), the rate of weight increase of the alloy slowed down. Figure 10b illustrates that during oxidation at 773 K and 823 K, there was a slight decline in the corresponding curves due to the decomposition of hydroxides on the alloy surface, but at 873 K, there was a significant decrease in these curves, resulting in a peak on the curve. Observing Figure 10c, at approximately 25 min of reaction time during oxidation at 773 K and 823 K, there was a gradual increase in alloy weight, indicating its isothermal oxidation behavior adheres to the parabolic kinetic law. As shown in Figure 10d, the alloy weight remained essentially constant between 100 to 180 min. Therefore, based on the above analysis, it can be inferred that the weight gain of the alloy mainly occurred during the heating phase. Additionally, the study also found that the initially formed MgO, Y_2_O_3_, Gd_2_O_3_, and ZnO oxides undergo substitution reactions later, ultimately forming a dense film composed of rare earth elements (Gd_x_, Y_2−x_)O_3_, inhibiting internal oxygen diffusion and enhancing the flame-retardancy performance of magnesium alloys.

**Figure 11:**
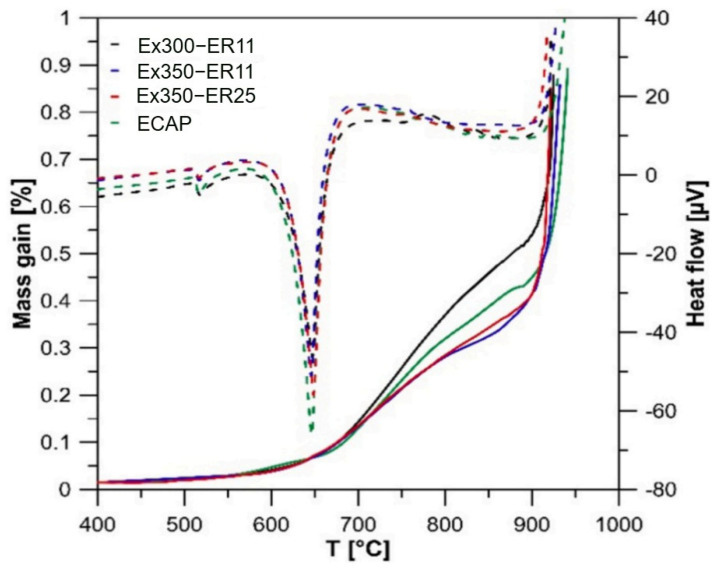
Thermogravimetry (TGA: solid line) and differential thermal analysis measurements (DTA: dashed line) [2].

Additionally, Y and Gd, as reactive elements, exhibit a synergistic effect when combined with Ca, simultaneously maintaining the alloy’s mechanical properties while augmenting its flame retardancy. Dvorsky et al. [81] observed that in the Mg-0.5Y, Mg-0.5Gd, Mg-0.5Ca, and Mg-0.5Y-0.5Gd-0.5Ca alloys, the collaborative action of Y, Gd, and Ca resulted in a corrosion rate better than that of binary systems, leading to a greater increase in ignition temperature. Building upon this synergistic effect, Kubásek et al. [9] developed a novel Mg-4.5Gd-3.4Y-2.6Ca alloy. At elevated temperatures, a protective layer comprising Y_2_O_3_, Gd_2_O_3_, and CaO formed on the alloy surface and effectively prevented oxidation, thereby elevating the alloy’s ignition temperature to 1373 K. Minárik et al. [2], in their thermal gravimetric and differential thermal analysis of the Mg-2Y-2Gd-1Ca alloy, depicted the dynamic curves illustrated in Figure 11. At 600 °C, a rapid decline in the alloy’s DTA curve (dashed line) indicated an endothermic reaction occurrence. Subsequently, a significant increase in the TGA curve slope was observed, attributed to the promotion of alloy oxidation at 600 °C, forming oxides based on Y and Gd (note: not the non-lightweight MgO) [2]. Around 1173 K, the TGA curve exhibited a sharp ascent, signifying an exothermic reaction, marking the onset of ignition and extensive oxidation. Further investigation revealed that Y and Gd possess the capability to foster the formation of a stable oxide film. At high temperatures, this film effectively impedes oxygen from contacting magnesium, elevating the ignition temperature of Mg from 923 K to 1223 K, thereby substantially enhancing the flame retardancy of magnesium alloys.

**Figure 12:**
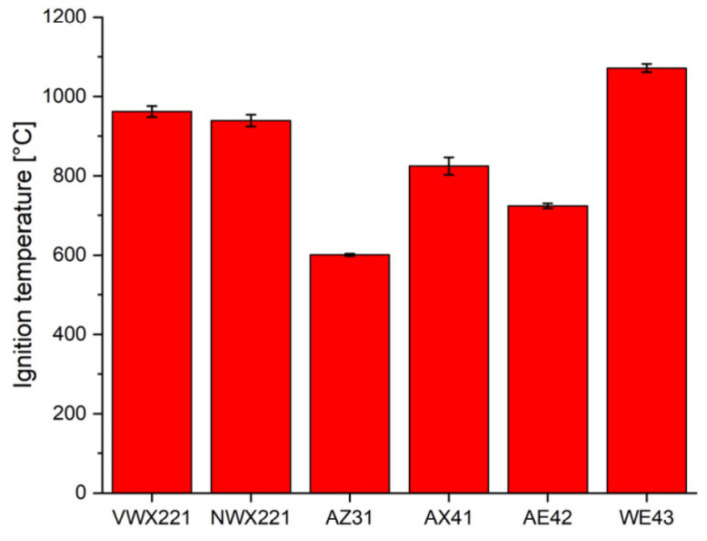
Ignition temperature of different magnesium alloys [82].

Figure 12 illustrates the ignition temperatures of the AZ31, AX41, AE42, WE43, VMX221 (Mg-2Gd-2Y-1Ca), and NWX221 (Mg-2Nd-1Y-1Ca) alloys [82]. It can be observed from the graph that the ignition temperatures for the WE43, VMX221, and NWX221 alloys are 1345 K, 1237 K, and 1213 K, respectively. However, the protective MgO oxide layer is lost at 873 K [29]. Consequently, the elevated ignition temperatures are attributed to oxides such as Nd_2_O_3_, Y_2_O_3_, Gd_2_O_3_, and CaO [60,61,83,84,85,86], which reinforce the alloy oxide layer, impeding the oxidation process of the alloy. For the WE43 alloy, the high content of rare earth elements is the fundamental cause for its higher ignition temperature. The lower ignition temperature of the AZ31 alloy is due to the inadequate protection of its aluminum and zinc components for an extended duration [36]. Furthermore, it is discovered that the dissolution level of secondary phase particles in VMX221 and NWX221 alloys can decelerate the alloy oxidation and ignition processes.

The flame-retardant performance of Mg alloys is closely associated with the addition of rare earth elements, unlike Mg-Ce alloys. Qian et al. [87] observed that in comparison to the Mg-5.46Gd-4.02Y-0.2Zr alloy, the Mg-5.3Gd-4.11Y-0.13Zr alloy, with higher Y content, forms a more compact and stable Y_2_O_3_ oxide film at elevated temperatures, thereby enhancing its flame-retardant properties. Dvorsky et al. [65] found that incorporating small amounts of rare earth elements significantly elevates the ignition temperature of magnesium alloys compared to pure Mg alloys (at 923 K). For instance, the ignition temperature of the Mg-0.5Y-0.5Gd-0.3Ca alloy can reach 1173 K. However, further addition of alloying elements has a minor impact on the ignition point of magnesium alloys. For instance, the Mg-2Y-2Gd-1Ca and Mg-4Y-4Gd-2Ca alloys exhibit remarkably similar ignition temperatures, both at approximately 1368 K.

The mechanism of action for Y and Gd in binary rare earth magnesium alloys is complex, and different oxidation environments significantly affect the flame-retardant properties of Y and Gd-containing rare earth magnesium alloys. Liu et al. [88] discovered that in a dry pure O_2_ environment, the Mg-Gd-Y-Zr alloy exhibits superior antioxidative properties compared to pure magnesium. However, in a moist O_2_ environment, due to the weakening effect of Y and Gd on the flame retardancy of the aforementioned alloy, combined with the ease of generating Mg(OH)_2_ in pure magnesium, the antioxidative properties of pure magnesium are better enhanced. It is evident that the flame-retardant performance of Y and Gd-containing magnesium alloys warrants further investigation.

#### 4.1.2. Nd and Dy

Rare earth elements often exhibit a synergistic effect among themselves. Ding et al. [89] discovered that the synergistic effect of Nd and Dy on the flame retardancy of the AZ91D alloy involves the formation of a dense oxide film composed of MgO, Nd_2_O_3_, and Dy_2_O_3_. Within a certain range, an increase in Nd content gradually elevates the alloy’s ignition temperature. Li et al. [90] delved deeper into the synergistic flame-retardant mechanism of Nd and Dy, revealing that at high temperatures, a compact oxide film composed of Dy_2_O_3_ and MgO forms. Additionally, the synergistic effect of Nd and Dy significantly reduces the content of the low-melting phase β-Mg_17_Al_12_ and alters the distribution of the low-melting phase’s grain boundaries from a continuous network to a dispersed form. This transformation prompts a reduction in oxygen diffusion channels, enhancing the flame retardancy of magnesium alloys. Hence, investigating the impact of rare earth element synergy on the flame retardancy of magnesium alloys from the perspective of intermetallic compounds is both feasible and effective. Therefore, further in-depth exploration of the synergistic mechanisms is necessary to develop magnesium alloys with excellent comprehensive performance.

#### 4.1.3. Y and Nd

**Figure 13:**
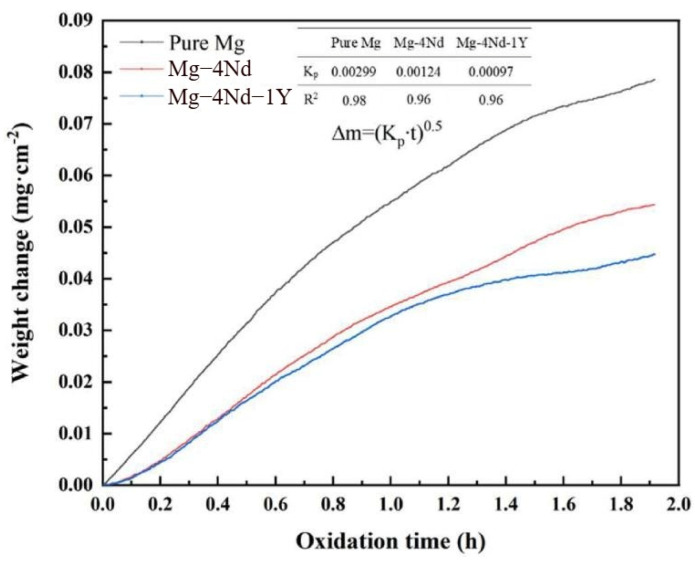
The weight change curves measured in Ar + 20%O_2_ at 500 °C for 2 h by TGA [92].

Yttrium (Y) and Neodymium (Nd) represent another effective combination of rare earth elements for enhancing the flame-retardant properties of magnesium alloys. Zhao et al. [91] discovered, under pyrolytic gasification conditions at 973 K using resin-sand, that the oxide layers of the Mg-4.32Y-2.83Nd-0.41Zr alloy primarily consisted of Y_2_O_3_ in the outer and inner layers, with an intermediate layer of MgO. Y_2_O_3_ particles tend to grow and segregate with prolonged oxidation time, hindering the diffusion of oxygen ions. This effectively impedes internal oxidation of the matrix, thereby enhancing the alloy’s flame retardancy. In a separate study, Wang et al. [92] conducted oxidation experiments for two hours at 500 °C in an Ar + 20%O_2_ environment on Mg-4Nd-1Y, Mg-4Nd, and pure Mg, as depicted in Figure 13 by their thermogravimetric curves. The results illustrate that compared to Mg-4Nd and pure Mg, the Mg-4Nd-1Y alloy exhibited a lower rate constant and a relatively gradual weight gain curve, suggesting superior oxidation resistance. This is attributed to the synergistic effect of Y and Nd, wherein Nd reduces the critical concentration of Y, enabling the formation of a dense Y_2_O_3_ oxide film even at lower Y concentrations.

#### 4.1.4. Y and Ce

The binary rare earth element combination of Y and Ce also demonstrates the capability to effectively enhance material properties, including antioxidation and corrosion resistance [93,94,95,96].

**Figure 14:**
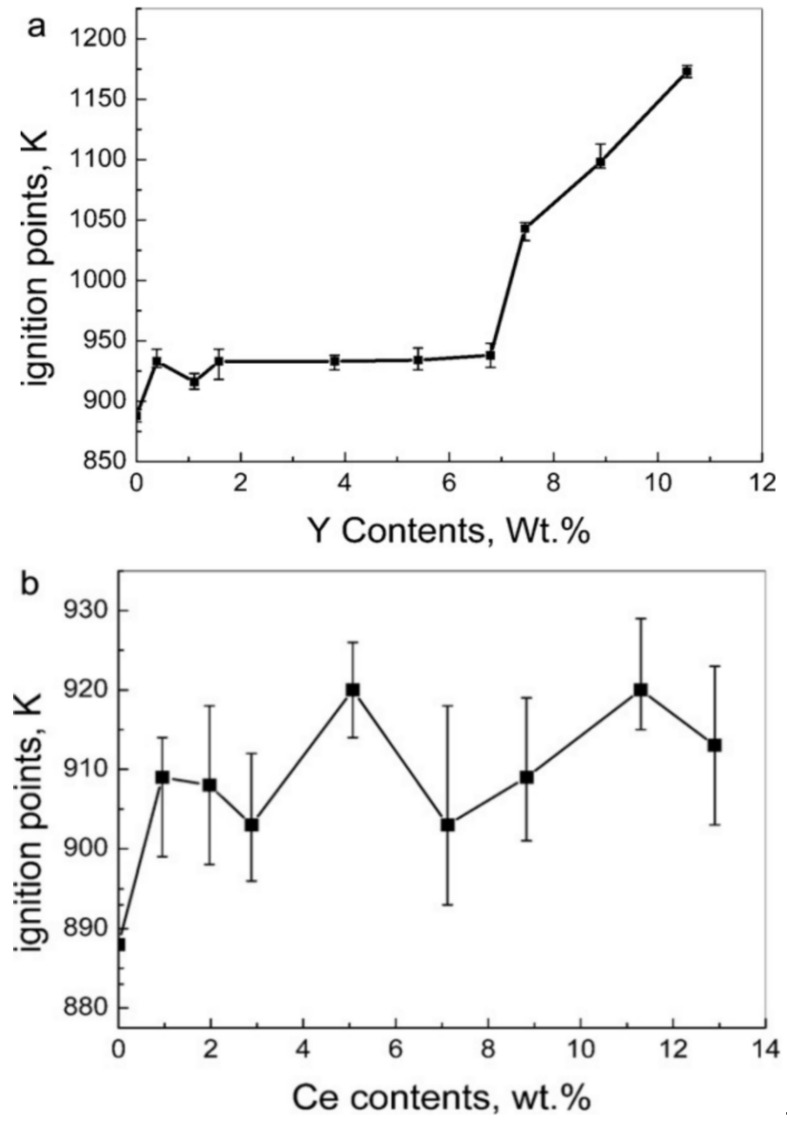

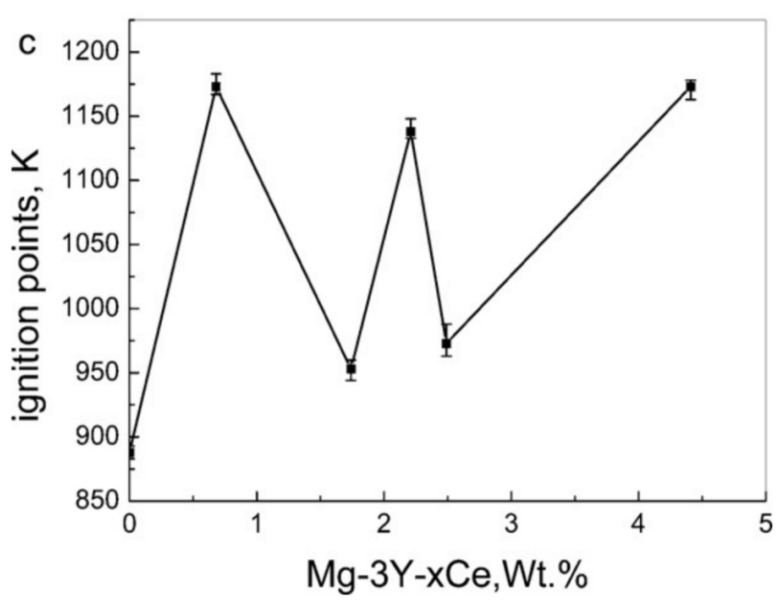
Ignition points of magnesium alloys with different compositions: (**a**) Mg-Y alloys; (**b**) Mg-Ce alloys; and (**c**) Mg-3Y-Ce alloys [100].

Adding rare earth elements like Y and Ce can enhance the antioxidation properties of magnesium alloys [97,98,99]. Fan et al. [100] conducted ignition tests on Mg-Y alloys by introducing Ce, as depicted in Figure 14. Upon observing Figure 14a, a threshold in Y content within Mg-Y alloys becomes evident: when Y content falls below 7 wt.%, the alloy’s ignition temperature remains unchanged; however, as Y concentration surpasses 7 wt.%, the ignition temperature of Mg-Y alloys notably escalates. Moreover, when Y concentration reaches 11 wt.%, the Mg-Y alloy sustains a non-combustible state for 0.5 h at 1173 K. Hence, it can be inferred that the alloy’s ignition temperature gradually rises with increasing Y content. Figure 14b illustrates that the individual addition of Ce has a minor impact on the ignition temperature of magnesium alloys. As portrayed in Figure 14c, introducing Ce alongside 3 wt.% Y significantly elevates the alloy’s ignition temperature, yet the ignition temperature remains unstable. However, incorporating 0.7 wt.% Ce into Mg-3Y alloys allows them to remain non-combustible for 0.5 h at 1173 K, attributed to the synergistic effect of Ce and Y substantially reducing the Y content while ensuring the alloy’s excellent flame-retardant properties. This phenomenon, also observed in other alloy systems, is termed the “third element effect.” Here, Ce acts as the third element, presumed to function as an “oxygen scavenger,” impeding oxygen diffusion into the alloy’s interior, thereby preventing internal oxidation of the alloy [101].

Guan et al. [102] discovered the instability of the ignition temperature in Mg-3Y-xCe alloys. The underlying reason lies in the inner layer of the oxide film formed at high temperatures, which impedes the outward diffusion of Y solute within the matrix, leading to a weakened ability of the outer oxide film to regenerate. Furthermore, from an electrochemical perspective, the addition of low-valence Ca^2+^ ions was found to reduce the speed of inward diffusion of oxygen ions, thereby slowing down the alloy oxidation process. Moreover, Bobryshev et al. [103] found that the addition of Ca elements in magnesium alloys resulted in a greater increase in the ignition temperature compared to Ce elements [104]. Zhou et al. [12], upon adding Ca to Mg-Y-Ce alloys, observed that among the oxide films of MgO, Y_2_O_3_, and CaO formed at high temperatures, only the PBR value of the Y_2_O_3_ oxide film exceeded 1. This indicates that Y_2_O_3_ contributes more significantly to the oxide layer than the other two, effectively preventing oxygen diffusion into the matrix, thereby endowing the alloy with better flame retardancy and non-flammability. Table 1 lists the ignition temperatures of pure Mg and various alloys. From the observations in Table 1, it is evident that with an increase in Y content, the rising trend of the ignition temperature in Mg-Y alloys gradually slows down. Additionally, upon adding 0.6 wt.% Ca, the Mg-3Y-0.6Ca alloy can sustain non-combustion at 1233 K for 0.5 h.

In summary, the analysis of the flame-retardant mechanism of Mg-Y-Ce alloys requires consideration from multiple perspectives and factors. Wang et al. [105] observed a significant influence of the addition ratio of Y and Ce on the ignition point of magnesium alloys. When the ratio of Y to Ce is 1.33, the ignition point of the Mg-2Y-1.5Ce-0.6Zr alloy reaches 920 °C, exhibiting optimal flame retardancy. However, in high-temperature environments, the outer layer of the oxide film formed by the aforementioned alloy consists of Y_2_O_3_ and MgO, while the inner layer comprises CeO_2_ and MgO. This unique structure effectively impedes the internal diffusion of oxygen, further enhancing the flame-retardant properties of the magnesium alloy beyond its original characteristics.

#### 4.1.5. Nd and Gd

Currently, there is limited research on the influence of rare earth elements Nd and Gd on the flame-retardant properties of magnesium alloys. The addition of Nd and Gd to Mg results in their high affinity for oxygen compared to magnesium. Consequently, they react initially with oxygen at high temperatures, forming rare earth oxides (Nd_2_O_3_ and Gd_2_O_3_) [106]. These rare earth oxides can restrict the oxidation of magnesium, thereby enhancing the alloy’s resistance to oxidation. Furthermore, a dual-phase MgO-Nd_2_O_3_ structure forms on the alloy’s surface, altering the alloy’s oxidation behavior, thus causing the heat-treated alloy to oxidize slower compared to the as-cast alloy.

### 4.2. Effect of Ternary Rare Earth on Flame Retardancy of Magnesium Alloys

#### Y, Nd, and Gd

Based on the synergistic effect, scholars have developed typical ternary rare earth magnesium alloys, primarily adding Yttrium (Y) and Neodymium (Nd). Liu et al. [107] measured the ignition temperature of the WE43 alloy at 917 K, while Kumar et al. [93] measured an increase in the ignition temperature of the WE43 alloy to 1003 K in air. This demonstrates the crucial role of oxygen molecules in the antioxidation process of magnesium alloys. Furthermore, rare earth elements Y, Gd, and Nd oxidize before Mg, forming a dense oxide film that prevents oxygen from contacting the substrate, thereby enhancing the alloy’s antioxidation properties [66,69,108,109,110].

It is evident that rare earth elements can enhance the flame-retardant performance of magnesium alloys because these elements generate highly thermally stable oxides within the alloy. These oxides form a barrier layer on the surface of the magnesium alloy, effectively inhibiting further reaction between oxygen and the alloy.

Wang et al. [111] observed a significant increase in elongation rate with increasing Gd content in Mg-5Y-3Nd-0.6Zr-xGd alloys, and the alloy’s heat-resistant phases notably increased with Gd addition. Luo et al. [112] found that the presence of Y_2_O_3_ significantly affects the mechanical properties of WE43 (Mg-Y-Gd-Nd-Zn-Zr) alloys. By replacing Y with Gd, it was found that low-Y alloys (Mg-2Y-3Gd-2Nd-0.5Zn-0.5Zr) and high-Y alloys (Mg-4Y-1Gd-2Nd-0.5Zn-0.5Zr) exhibit similar mechanical properties at high temperatures, providing direction for replacing high-Y content Mg-Y-RE alloys. Liu [113] and others also discovered that after high-temperature oxidation, the ultimate compressive strength and tensile strength of the WE43 alloy decreased. However, the oxide layer formed by oxygen and Yttrium enhanced the alloy’s surface hardness, wear resistance, and ductility. Thus, the significant improvement in the mechanical properties of ternary rare earth magnesium alloys provides new avenues for developing a new generation of ternary rare earth flame-retardant magnesium alloys.

## 5. Summaries and Future Development

In the past decade, the flame retardancy of magnesium alloys has emerged as a prominent research focus in the aerospace industry. A review of the aforementioned research progress reveals significant advancements in enhancing the flame-retardant properties of magnesium alloys. However, lingering challenges persist without resolution:

(1) The protective nature of the oxide film depends on its structure, with the PBR model serving as a commonly used evaluation model for oxide film protective capability. Nevertheless, it has certain limitations, lacking PBR values for different matrix alloys and overlooking the influence of alloy elements on factors such as solid solution strengthening, grain refinement, and mechanical properties of the oxide film. Therefore, further development and supplementation of flame-retardancy models are crucial.
(2) The second phase can influence the composition and structure of oxides, thereby indirectly affecting the antioxidation and flame-retardant properties of magnesium alloys. Improving the microstructure of alloys through rapid solidification technology effectively enhances the flame retardancy of alloys. However, besides the melting point, the size and volume fraction of the second phase also significantly impact the oxidation rate of the matrix. It is necessary to explore new technologies for comprehensively improving the microstructure of alloys. Additionally, research on the oxidation sequence of alloys is scarce.
(3) The addition of specific alloying elements such as Ca, Be, and Sr contributes to an increase in the ignition point of alloys. Apart from the type of elements, their content is also crucial. However, there is limited research on the impact of various specific element addition amounts and optimal ratios on the flame-retardant performance of magnesium alloys.
(4) Adding binary and ternary rare earth elements can form a stable rare earth oxide protective layer on the alloy surface. However, studies on the synergistic effects of binary or higher rare earth elements and other elements like Ca on the flame-retardant performance of magnesium alloys are scarce. It is essential to develop novel rare earth magnesium alloys with comprehensive properties to enhance the safety of magnesium alloys in aviation applications.

Therefore, for a more accurate evaluation and improvement of the high-temperature oxidation behavior and flame-retardant capability of magnesium alloys, establishing a more comprehensive and in-depth theoretical framework in future research should focus more on the integrated impact of ternary and higher rare earth elements on the flame-retardant performance of magnesium alloys, as well as the interactions between different factors. It is anticipated that innovative flame-retardant magnesium alloy materials will emerge in the future.

## Figures and Tables

**Table 1:** Ignition temperatures of pure Mg and various alloys [12].

Composition/wt.%	Ignition Point/K
Mg	933
AZ31	892
Mg-1.5Y	977
Mg-3Y	1091
Mg-6Y	1133
Mg-10Y	1169
Mg-5Y-0.1Ca	1153
Mg-5Y-0.3Ca	1213
Mg-5Y-0.6Ca	No ignition
Mg-4Y-0.1Ca	1109
Mg-4Y-0.5Ca	No ignition
Mg-4Y-0.9Ca	No ignition
Mg-3Y-0.6Ca	No ignition
Mg-3Y-0.4Ca-0.2Ce	977
Mg-3Y-0.5Ca-0.2Ce	1091
Mg-3Y-0.8Ca-0.2Ce	1133
Mg-3Y-1.2Ca-0.2Ce	1169

## Data Availability

The raw data supporting the conclusions of this article will be made available by the authors on request.
